# Design of an Active–Passive Composite Impedance Controller for a Soft Robotic Arm Under Contact Constraints

**DOI:** 10.3390/biomimetics9120742

**Published:** 2024-12-05

**Authors:** Bo Yan, Yinglong Chen, Cheng Zhou, Qiang Sun, Fei Gao, Xinyu Yang, Xingtian Xiao

**Affiliations:** 1 The Naval Architecture and Ocean Engineering College, Dalian Maritime University, Dalian 116026, China; yanbo@dlmu.edu.cn (B.Y.); s2549724961@163.com (Q.S.); gaofei@dlmu.edu.cn (F.G.); yang2024@dlmu.edu.cn (X.Y.); xiao2024@dlmu.edu.cn (X.X.); 2 Wuhan 2nd Ship Design a Research Institute, Wuhan 430205, China; webmail01@163.com

**Keywords:** active–passive composite impedance, passive impedance characteristics, soft robotic arm

## Abstract

The inherent passive impedance characteristics of soft robotic arms provide excellent environmental adaptability. When a soft robotic arm interacts with its surroundings, its passive impedance responds swiftly, preventing rigid collisions that could damage the arm and ensuring high safety. However, during the movement of the soft robotic arm, these passive impedance properties are uncontrollable, making it impossible to achieve precise impedance control in constrained environments by relying solely on passive mechanisms. Therefore, this paper integrated active impedance control with the passive impedance characteristics of soft robotic arms, proposing an active–passive composite impedance controller. Additionally, a position-based impedance controller was designed for comparative analysis. Finally, this article developed both control systems and conducted simulations and experiments, demonstrating that the composite active–passive impedance controller offers superior control performance and environmental adaptability.

## 1. Introduction

In recent years, soft robots have become a major research focus within robotics [1,2,3,4,5]. The scope of soft robots recognized by the International Federation of Robotics include the following two points: firstly, from a material perspective, the robot body should be made of flexible materials; secondly, from a functional perspective, it is allowed for the robot’s body to have rigid components, but the different components within the robot should exhibit behavior characteristics similar to soft bodied organisms through mutual cooperation. With exceptional shape-morphing capabilities, soft robots can adapt their form to suit environmental conditions, demonstrating excellent adaptability. At present, soft robots can be divided into soft worms [6], soft robotic fish [7], soft octopuses [8,9], and soft robotic arms based on biomimetic principles. Among these, soft robotic arms represent a critical area of study. Soft robotic arms feature unlimited degrees of freedom and flexible structures, enabling them to handle delicate objects and operate in complex, unstructured environments [10,11,12,13,14]. This adaptability gives soft robotic arms great potential in fields such as industry, medical surgery, and defense.

As soft robotic arms can interact safely with their surroundings, enhancing the controllability of contact forces between the arm and its environment has become a crucial research focus. Several scholars have explored force/position hybrid control for soft robotic arms. Bajo et al. developed a force/position hybrid control framework for continuum robots [15]. Additionally, Yip et al. proposed a model-free control method, where the robot’s Jacobian matrix is estimated in real time based on actuator and end-effector measurements and fed back to the task space for closed-loop control [16]. This hybrid control approach, however, has limitations, requiring frequent switching during operation and being applicable only to decoupled position/force tasks. In contrast, soft robotic arms often operate in constrained environments, where they must safely interact with surrounding obstacles, necessitating that basic tasks be completed under coupled position/force constraints [17].

To stabilize both the desired motion and interaction forces during task execution, some researchers have introduced impedance control into soft robotic arm control. Impedance control converts the dual control objectives of force and position into a dynamic relationship between external forces and robotic motion, enabling stable handling of tasks involving coupled position/force constraints. Hogan systematically introduced the concept of impedance control in 1985, laying the groundwork for force/position control in robotics [18,19]. Impedance control describes the dynamic relationship between a robot’s displacement and contact forces, often modeled as a second-order spring–mass–damper system [20]. Impedance controllers are categorized into position-based and force-based types. Position-based impedance controllers establish a model linking the end-effector’s displacement to contact forces, with dynamic adjustment achieved by tuning parameters like inertia, damping, and stiffness. In contrast, force-based impedance controllers modify the robot’s dynamics to match a target impedance model by adjusting control inputs, allowing fine-tuned control over impedance characteristics. Given its significance for human–robot interaction, impedance control has been extensively studied for rigid robots, including industrial robots [21], inspection robots [22], and rehabilitation robots [23], over the past several decades.

Furthermore, compliant robots share similarities with soft robots. A compliant robot is a robot with a certain degree of compliance, which can be achieved through various means, such as using compliant materials, compliant joints, or force sensors, to reduce impact and damage when interacting with the environment. The main differences between the two lie in their material properties, deformation capabilities, and application scenarios. Soft robots use softer materials and have stronger deformation capabilities, making them more suitable for scenarios that require high flexibility, such as medical surgery. In contrast, compliant robots can achieve compliance in various ways and have a broader range of applications, such as in assembly, polishing, and other fields. In the field of robot control, besides the aforementioned tasks, compliant control is also a control method for achieving safe, stable, and efficient interaction between robots and the environment. Ding et al. provided a comprehensive review of the mechanical design of compliant robots, which includes relevant content such as compliant control [24]. This has a certain reference value for our understanding and research on the control of soft robots. Ding et al. proposed an optimization method based on FEA to optimize the constant force characteristics of a flexible constant force module [25]. This research provides important insights for the optimization design of compliant mechanisms and has a certain reference value for this paper in considering force control and performance optimization of soft robotic arms.

In the field of soft robotics, as noted in a review article [26], there remains limited research on impedance control. Toscano et al. proposed an impedance control method for single-segment soft robotic arms based on kinematic and dynamic models, eliminating the need to measure or estimate contact forces [27]. Santina et al. introduced a Cartesian space impedance controller for continuum robots with constant stiffness and damping [28]. He et al. developed a task-space variable impedance control approach for continuum robots and demonstrated closed-loop stability using a novel Lyapunov function [29]. Su et al. presented a Cartesian-space coordinated variable impedance controller for cable-driven continuum robots [30]. Mazare et al. proposed a joint-space variable impedance control strategy for modular soft robots, combining adaptive inverse sliding mode control with joint-space load estimation [31]. Seleem et al. developed a learning-based motion planning method and dynamic impedance controller, enabling dual-segment continuum robots to perform learning, planning, and trajectory tracking in dynamic environments [32].

**Figure 1:**
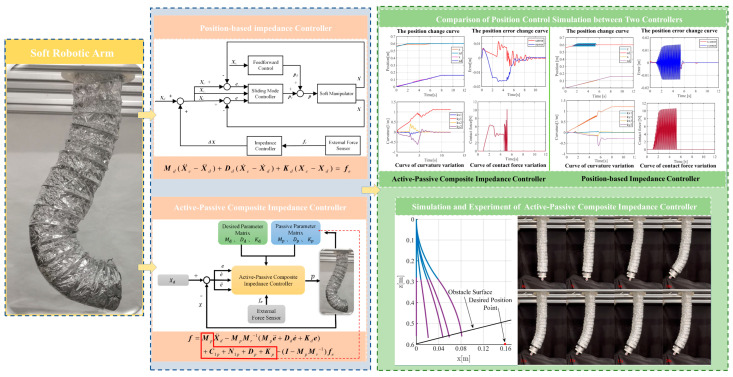
Research roadmap of this article.

As the above review indicates, compared to the advancements in impedance control for rigid robots, research on impedance control for soft robotic arms remains relatively limited. Current studies primarily focus on the design of active impedance controllers, with less emphasis on utilizing the passive impedance properties inherent to soft robotic arms. The natural compliance of soft robotic arms (passive impedance characteristics) provides strong environmental adaptability, allowing rapid response to contact and preventing damaging rigid collisions, which enhances safety. Therefore, to effectively and controllably leverage these passive impedance properties, this study explores composite active–passive impedance control for soft robotic arms. Based on force impedance control, this paper integrates the inherent passive impedance characteristics of soft robotic arms to design a composite controller. To validate and compare the control performance, a position-based impedance controller is also developed. The design processes for both controllers are detailed in the following chapters. The overall structure of the text is shown in Figure 1.

The motivation of this study lies in the fact that while the passive impedance characteristics of a soft robotic arm provide excellent environmental adaptability during interaction with the environment, they are uncontrollable and cannot achieve precise impedance control in constrained environments relying solely on passive mechanisms. Therefore, it is necessary to explore a method that combines active and passive impedance control to effectively utilize the passive impedance properties of the soft robotic arm while achieving precise control.

The innovation of this study is the development of a composite active–passive impedance controller that integrates the passive impedance characteristics of the soft robotic arm with active impedance control. By designing this controller, the soft robotic arm can exhibit improved compliance and stability when in contact with obstacles, reduce positional error and contact force, avoid jamming, and respond rapidly to external disturbances in complex environments. Compared with traditional position-based impedance controllers, the composite active–passive impedance controller offers superior control performance and environmental adaptability.

Soft robotic arms have a wide range of potential applications, especially in fields that require high flexibility, adaptability, and safety. For example, in minimally invasive surgery, soft robotic arms can perform precise operations within narrow and complex body cavities, avoiding damage to surrounding tissues. Additionally, in the event of a disaster, soft robotic arms can enter complex rubble environments to carry out search and rescue tasks, precisely avoiding obstacles and completing the mission. Their strong adaptability allows them to perform rescue operations in irregular terrains. Therefore, research on the control of soft robots under contact constraints is of great practical significance, as it can provide new solutions for future applications such as minimally invasive surgery and search and rescue operations in complex environments.

The relevant parameters of the soft robotic arm and the hydrostatic drive system mathematical model in this paper are shown in Table 1.

## 2. Composite Active–Passive Impedance Control Strategy

### 2.1. Composite Active–Passive Impedance Controller

**Figure 2:**
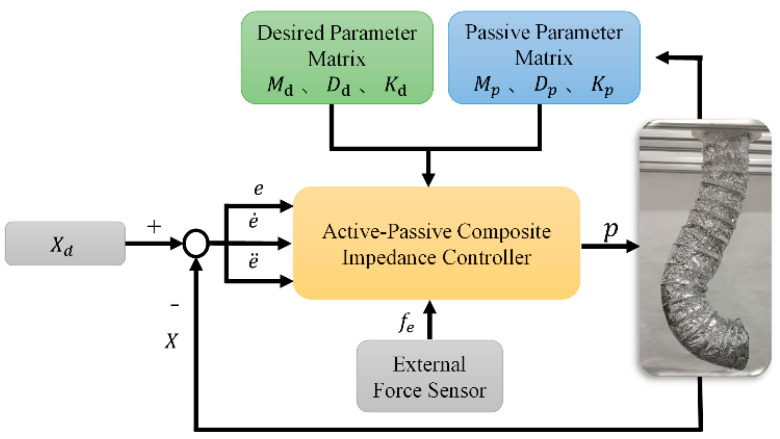
Active–passive composite impedance control block diagram.

Based on the impedance control model introduced in Section 1, force-based impedance controllers differ from position-based controllers by requiring access to the robot’s internal force control loop. This approach necessitates an understanding of the robot’s dynamic model and characteristics, allowing the design of a control input, τ, to transform the dynamic model into a desired target impedance model that exhibits the required impedance behavior. In this subsection, the inherent passive impedance characteristics of the soft robotic arm are incorporated into the design of a force-based impedance controller, creating a composite active–passive impedance controller. The control principle is illustrated in Figure 2.

As shown in the control block diagram in Figure 1, the composite active–passive impedance controller considers both the desired impedance parameters and the passive impedance characteristics of the soft robotic arm. To analyze the inherent passive impedance characteristics of the soft robotic arm, the analysis can be started with its dynamic model. In previous work, the joint-space dynamic equations for the soft robotic arm were developed [33]. In previous work, the first segment of the soft robotic arm is defined as consisting of soft units 1, 2, and 3, while the second segment consists of soft units 4, 5, and 6. When performing dynamic analysis of the soft robotic arm, the posture of the *i*-th segment can be described by the length li along the axis of the soft arm, as well as the bending strains *k_xi_, k_yi_,* and *k_zi_* around the coordinate axes *x_i_*, *y_i_*, and *z_i_* of the corresponding segment’s coordinate system. Therefore, the motion variable matrix for the *i*-th segment can be represented as: [*l_i_ k_xi_ k_yi_ k_zi_*]. By treating the motion variables of each segment as state variables in the dynamic computation process, the joint-space state variable ***q*** can be expressed as:

(1) $$\begin{array}{l} q={\left[\begin{array}{cccc} q_{1} & \begin{array}{cc} \cdots & q_{i} \end{array} & \cdots & q_{n} \end{array}\right]}^{T}\\ q_{i}={\left[\begin{array}{cccc} l_{i} & k_{xi} & k_{yi} & k_{zi} \end{array}\right]}^{T} \end{array}$$

In the equation, ***q**i* represents the state variable matrix of the *i*-th segment of the soft robotic arm, where *i∈ 1, 2,…,n.*

However, to emphasize the influence of passive impedance when the soft robotic arm interacts with its environment, the joint-space dynamics are modified through specific procedures so that its passive impedance matrix can be explicitly included. The revised dynamic equation is presented as follows:

(2) $$M\left(q\right)\ddot{q}+C_{1}\left(q,\dot{q}\right)\dot{q}+D\dot{q}+Kq+N_{1}\left(q\right)=\tau +\tau_{e}$$

In the equation, ***M*** (***q***) represents the inertia matrix, and its expression is shown as follows:

(3) M(q)=mh∂Hi  i−1∂qαXi  i−1×mh∂Hi  i−1∂qα

$C_{1}\left(q,\dot{q}\right)$ denotes the Coriolis matrix, ***D*** is the damping matrix, $C(q,\dot{q})$ is the sum of $C_{1}\left(q,\dot{q}\right)$ and ***D***, and its expression is shown as follows:

(4) C(q,q˙)=hm∂2Hi  i−1∂q∂q˜q˜˙+dv∂Hi  i−1∂qαXi  i−1×hm∂2Hi  i−1∂q∂q˜q˜˙+dv∂Hi  i−1∂qα

***K*** represents the stiffness matrix, which reflects the elastic properties of the soft robotic arm. ***N***_1_ is a composite matrix that includes gravitational forces and interaction forces between adjacent segments of the soft robotic arm. ***N*** (***q***) is the sum of ***N***_1_ and ***K***, and its expression is shown as follows:

(5) Nq=fi,e  i−1Mi,e  i−1−R0  i00GnTXi  i−1×R0  i00GnT−Ri  i−1fi+1  iXi  i−1×Ri  i−1fi+1  i+Ri  i−1Mi+1  i

***τ*** represents the driving force of the soft robotic arm, while ***τ****_e_* denotes the contact force exerted by the external environment; its expression is shown as follows:

(6) τ=fi,d  i−1Mi,d  i−1

As shown in Figure 1, the feedback information required for the control system of the soft robotic arm, such as position and contact force, is in the task space. Therefore, it is necessary to establish the task-space dynamic equation for the soft robotic arm. Using the method for deriving task-space dynamics from reference [34], the joint-space dynamic equation is transformed into the task-space dynamic equation. Let the end-effector position of the soft robotic arm be defined as ***X*** = [*x*, *y*, *z*]*^T^*, allowing us to establish the mapping between the end-effector position and the soft robotic arm’s shape parameters as ***X*** = *f_qx_* (***q***). Taking the first and second derivatives of this mapping yields:

(7) $$\begin{array}{c} \dot{X}=J(q)\dot{q}\\ \ddot{X}=J(q)\ddot{q}+\frac{d(J(q))}{dt}\dot{q} \end{array}$$

In the equation, ***J***(***q***) represents the Jacobian matrix between the task space and the joint space:

(8) J(q)=∂fgx(q)∂q=∂Tn,(1,4)  0∂q11∂Tn,(1,4)  0∂q12⋯∂Tn,(1,4)  0∂qn4∂Tn,(2,4)  0∂q11∂Tn,(2,4)  0∂q12⋯∂Tn,(2,4)  0∂qn4∂Tn,(3,4)  0∂q11∂Tn,(3,4)  0∂q12⋯∂Tn,(3,4)  0∂qn4  J(q)∈ℝ3×4n

By combining Equations (2) and (7), the following can be obtained:

(9) $$\ddot{X}=J(q)M{\left(q\right)}^{-1}(\tau +\tau_{e})-J(q)M{\left(q\right)}^{-1}(C_{1}(q,\dot{q})\dot{q}+D\dot{q}+Kq+N_{1}(q))+\frac{d(J(q))}{dt}\dot{q}$$

Following the task-space dynamic model derivation method in reference [34], the matrices in the joint-space dynamic model of the soft robotic arm are rewritten as follows:

(10) $$\begin{array}{l} \tau =J{\left(q\right)}^{T}f\begin{array}{c} \;\;f\in {\mathbb{R}}^{3\times 1} \end{array}\\ \tau_{e}=J{\left(q\right)}^{T}f_{e}\begin{array}{c} \;\;f_{e}\in {\mathbb{R}}^{3\times 1} \end{array}\\ M_{p}={\left(J(q)M{\left(q\right)}^{-1}J{\left(q\right)}^{T}\right)}^{-1}\begin{array}{c} \;\;M_{p}\in {\mathbb{R}}^{3\times 3} \end{array}\\ C_{1p}=M_{p}[J(q)M{\left(q\right)}^{-1}C_{1}(q,\dot{q})\dot{q}-\frac{d(J(q))}{dt}\dot{q}]\begin{array}{c} \;\;C_{1p}\in {\mathbb{R}}^{3\times 1} \end{array}\\ D_{p}=M_{p}J(q)M{\left(q\right)}^{-1}D\dot{q}\begin{array}{c} \;\;D_{p}\in {\mathbb{R}}^{3\times 1} \end{array}\\ K_{p}=M_{p}J(q)M{\left(q\right)}^{-1}Kq\begin{array}{c} \;\;K_{p}\in {\mathbb{R}}^{3\times 1} \end{array}\\ N_{1p}=M_{p}J(q)M{\left(q\right)}^{-1}N_{1}(q)\begin{array}{c} \;\;N_{1p}\in {\mathbb{R}}^{3\times 1} \end{array} \end{array}$$

Substituting the above transformations into the joint-space dynamic equation, the task-space dynamic equation for the soft robotic arm is derived as follows:

(11) $$M_{p}\ddot{X}+C_{1p}+N_{1p}+D_{p}+K_{p}=f+f_{e}$$

After the task-space dynamic model for the soft robotic arm is established, the design of the composite active–passive impedance controller proceeds. First, the target spring–mass–damper system equation is formulated as follows:

(12) $$\begin{array}{c} (M_{a}+M_{r})\ddot{e}+(D_{a}+D_{r})\dot{e}+(K_{a}+K_{r})e=f_{e}\\ M_{d}\ddot{e}+D_{d}\dot{e}+K_{d}e=f_{e}\\ e=X-X_{d} \end{array}$$

In this equation, ***M***_a_ represents the passive inertia matrix of the soft robotic arm, with ***M****_a_* = ***M**_p_*; ***D***_*a*_ is the passive damping matrix; ***K***_*a*_ denotes the passive stiffness (elasticity) matrix; ***M***_*r*_ is the active mass matrix; ***D***_*r*_ is the active damping matrix; ***K***_*r*_ is the active stiffness matrix; ***M***_*d*_ is the desired mass matrix; ***D***_*d*_ is the desired damping matrix; ***K***_*d*_ is the desired stiffness matrix; and f represents the driving force of the soft robotic arm, while $f_{e}$ denotes the contact force exerted by the external environment.

By combining Equations (11) and (12), the required composite active–passive impedance controller can be designed. The controller equation is shown as follows:

(13) $$\begin{array}{c} f=M_{a}{\ddot{X}}_{d}-M_{a}M_{r}^{-1}(M_{a}\ddot{e}+(D_{a}+D_{r})\dot{e}+(K_{a}+K_{r})e)\\ +C_{1p}+N_{1p}+D_{p}+K_{p}-(I-M_{a}M_{r}^{-1})f_{e} \end{array}$$

Since ***D**_d_ = **D**_a_ + **D**_r_*, ***K**_d_= **K**_a_ + **K**_r_*, ***M**_a_ = **M**_p_*, the controller can be expressed as:

(14) $$\begin{array}{l} f=M_{p}{\ddot{X}}_{d}-M_{p}M_{r}^{-1}(M_{p}\ddot{e}+D_{d}\dot{e}+K_{d}e)\\ +C_{1p}+N_{1p}+D_{p}+K_{p}-(I-M_{p}M_{r}^{-1})f_{e} \end{array}$$

### 2.2. Position-Based Impedance Controller

**Figure 3:**
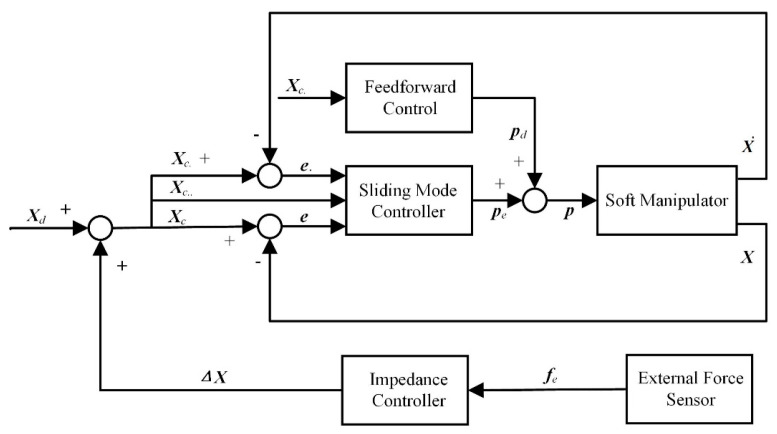
Position-based impedance control block diagram.

In the previous section, a composite active–passive impedance controller was designed. To compare and verify the superior control performance of this controller, a position-based impedance controller will be designed in this section. The control schematic is shown in Figure 3. This schematic consists of a dual-loop structure, including a position control inner loop and a force control outer loop, which is widely used in the field of robotic impedance control.

As shown in the control schematic in Figure 3, the position-based impedance controller consists of a position control inner loop and a force control outer loop. The force control outer loop is represented by the target impedance control model, as shown in Equation (15).

(15) $$M_{d}({\ddot{X}}_{c}-{\ddot{X}}_{d})+D_{d}({\dot{X}}_{c}-{\dot{X}}_{d})+K_{d}(X_{c}-X_{d})=f_{e}$$

In this equation, ***X****_d_* represents the initial desired position that the soft robotic arm needs to reach. When the soft robotic arm makes contact with environmental obstacles, the contact force is measured by force sensors and fed back to the impedance controller. Under the influence of the impedance controller, a position offset *Δ**X*** is generated, leading to a new desired position ***X****_c_*. The new desired position is then input into the inner-loop controller, where a sliding mode controller with feedforward compensation designed in previous work is used. [35]. The inner-loop controller ensures the tracking of the corrected desired position ***X****_c_*. Since ***X****_d_* is defined as the initial desired position the soft robotic arm needs to reach, rather than its starting position, if no contact force is applied during the movement (i.e., no position offset *Δ**X*** is generated), then ***X****_d_* will be equal to ***X****_c_*. Alternatively, when the external contact force is removed, the controller’s task will revert to tracking the initial desired position ***X****_d_* by the controlled displacement ***X***.

From the analysis of the impedance controller above, it is clear that the most important factor in impedance control is selecting appropriate target parameter matrices to regulate the dynamic relationship between displacement corrections and external contact forces, thereby achieving the desired impedance control effect. For the parameter matrices in the target impedance control model, the inertia matrix ***M****_d_* indicates that the soft robotic arm will exhibit greater inertia when experiencing higher acceleration. The larger the rotational speed of the soft robotic arm, the greater the effect of the damping matrix ***D****_d_*, which directly influences the oscillatory convergence rate of the control system. The stiffness matrix ***K****_d_* reflects the spring-like elasticity when the soft robotic arm deviates from the desired target position. These parameter matrices can be adjusted by selecting different values based on specific control objectives and working environments, thereby tuning the impedance characteristics of the soft robotic arm. Therefore, the proper selection of parameter matrices is crucial to the performance of the impedance control system.

## 3. Simulation Analysis

### 3.1. Comparison of the Control Performance Between the Active-Passive Composite Impedance Controller and the Position-Based Impedance Controller

After the two impedance controllers are designed, the control systems for both controllers were built in Simulink according to their control block diagrams. A comparative analysis of the control performance of the composite active–passive impedance controller and the position-based impedance controller was then be conducted through simulations. First, the position errors in the simulation and experimental process were defined: the absolute position error represents the difference between the actual measured position and the desired position of the soft robotic arm’s end in a given direction; the relative position error is the ratio of the absolute position error to the desired position of the soft robotic arm in that direction; the peak position error refers to the maximum position error during the soft robotic arm’s motion; the steady-state position error represents the position error when the soft robotic arm moves steadily after making contact with an obstacle; the peak contact force refers to the maximum contact force when the soft robotic arm makes contact with the obstacle; the steady-state contact force is the contact force when the soft robotic arm moves steadily after contact with the obstacle.

In the control research of soft robotic arms, response time, position error, and contact force with the external environment are crucial evaluation metrics. Response time determines the speed at which the soft robotic arm reacts to control commands. Its rapid response can prevent operational errors and collisions in various scenarios, reflecting the real-time reaction and effectiveness of the control system. Position error is a key indicator of the control accuracy of the soft robotic arm. Precise position control greatly impacts the success of many tasks, as it relates to the safety of interaction between the soft robotic arm and the environment, and reflects the accuracy of the kinematic model and the controller’s ability to resist interference. Finally, the contact force between the soft robotic arm and the external environment is also an important performance metric. In tasks such as human–robot collaboration and rescue operations, properly controlling contact force can prevent injury and damage, helping to improve operational stability and reliability. In impedance control, contact force is used as a feedback signal to adjust the robot’s position, velocity, and motion mode. Therefore, in subsequent simulations and experiments, position error, response time, and contact force are selected to evaluate the control performance of the controller.

**Figure 4:**
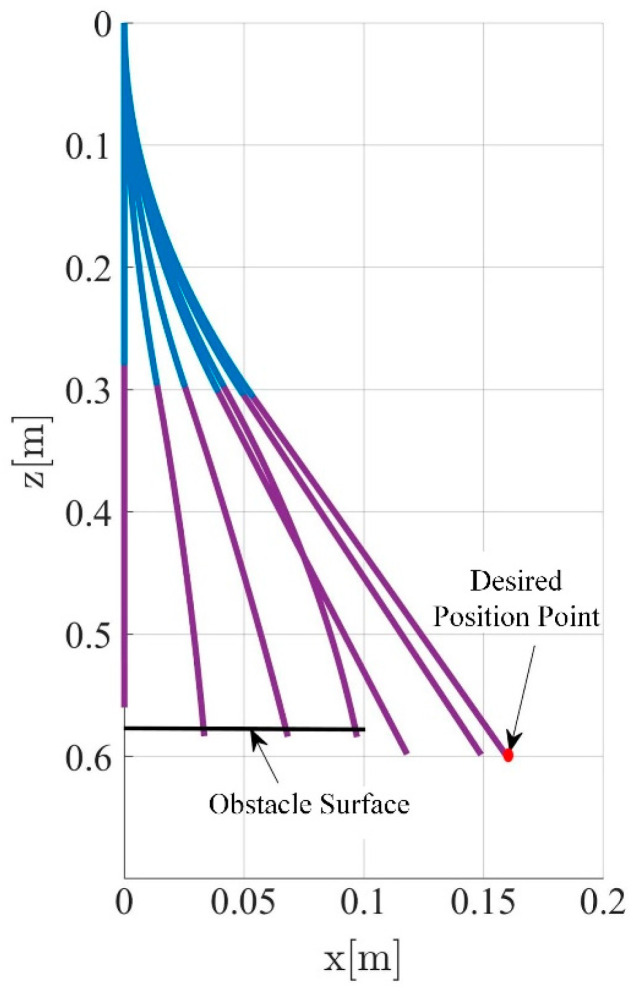
Motion trajectory with an obstacle surface. ( The blue line represents the first segment of the soft joint, and the purple line represents the second segment of the soft joint.)

**Figure 5:**
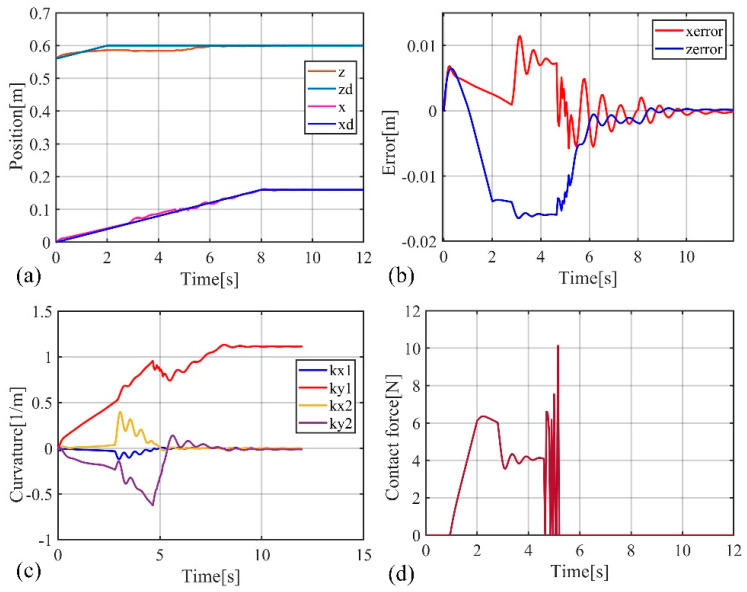
Simulation results of the active–passive composite impedance controller, (**a**) The position change curve of the end of the soft robotic arm in the x and z directions; (**b**) The position error change curve of the end of the soft robotic arm in the x and z directions; (**c**) Curve of curvature variation of soft robotic arm; (**d**) Curve of contact force variation at the end of a soft robotic arm.

To facilitate the simulation, the soft robotic arm will be controlled to move within the x-o-z plane. To highlight the control performance of the two controllers, a distinct working condition is set up in the simulation, where the soft robotic arm undergoes a complete process of free movement, contact with an obstacle, disengagement, and free movement again. The motion trajectory is shown in Figure 4. The desired position of the soft robotic arm’s end point is set as (0.16 m, 0.6 m) and an obstacle plane is placed at z = 0.58. The length of the obstacle plane is 0.1 m, and the stiffness of the obstacle surface is set to 1000 N/m. The simulation results using both impedance controllers are shown in Figure 4 and Figure 5.

**Figure 6:**
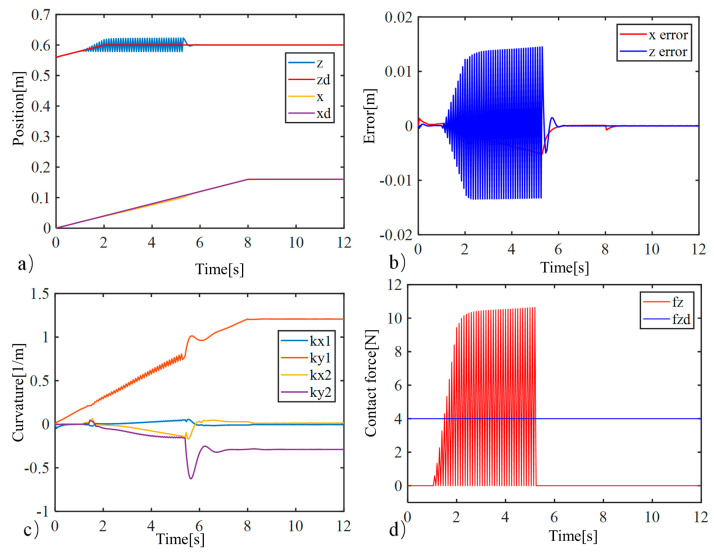
Simulation results of position-based impedance controller, (**a**) The position change curve of the end of the soft robotic arm in the x and z directions; (**b**) The position error change curve of the end of the soft robotic arm in the x and z directions; (**c**) Curve of curvature variation of soft robotic arm; (**d**) The contact force variation curve of the soft robotic arm end in the x and z directions.

Comparing Figure 5 and Figure 6, it can be observed that due to the obstruction of the obstacle, the motion trajectories of the soft robotic arm caused by both impedance controllers deviate from the desired path, resulting in motion errors and contact forces. The impedance characteristics introduced by the controllers allow the soft robotic arm to move compliantly along the obstacle surface without rigid collisions or jamming. Once the obstruction from the surface disappears, the contact force vanishes, and the soft robotic arm moves back to the desired position under the controller’s influence. In both cases, the position error and contact force stabilize at zero, indicating that once the obstacle is no longer present, both controllers successfully guide the soft robotic arm to the target position. Furthermore, by comparing Figure 5b–d and Figure 6b–d, it can be seen that smaller fluctuations in position and error during contact scenarios result from the active–passive composite impedance controller, with a smoother motion maintained and no significant changes in contact force. On the other hand, the position-based impedance controller exhibits larger fluctuations in position and error, failing to maintain smooth motion and producing more dramatic variations in contact force. From this comparison, it is clear that the position-based impedance controller does not ensure stable motion and produces significant position errors and contact force changes, whereas the active–passive composite impedance controller ensures more stable control performance and higher precision, showing a distinct advantage.

### 3.2. Control Performance of Active–Passive Composite Impedance Controller Under Multiple Complex Conditions

In the previous section, the control performance of the composite active–passive impedance controller and the position-based impedance controller under a flat obstacle condition was compared and analyzed. The simulation results show that the active–passive composite impedance controller designed in this paper outperforms the traditional position-based impedance controller. In this section, to further validate the superior control performance of the composite active–passive impedance controller, the method of controlling variables is used to keep other simulation conditions the same while varying the form of the obstacle surface, and the controller’s performance under different working conditions is analyzed. The specific simulation conditions are defined as follows: the soft arm is controlled to move in the x-o-z plane and the desired position of its end-effector is set to always be (0.16 m, 0.6 m). The obstacle surfaces are defined as flat, inclined, and arc-shaped respectively, and the active–passive composite impedance controller is used to control the movement of the soft robotic arm to obtain position and contact force data.

**Figure 7:**
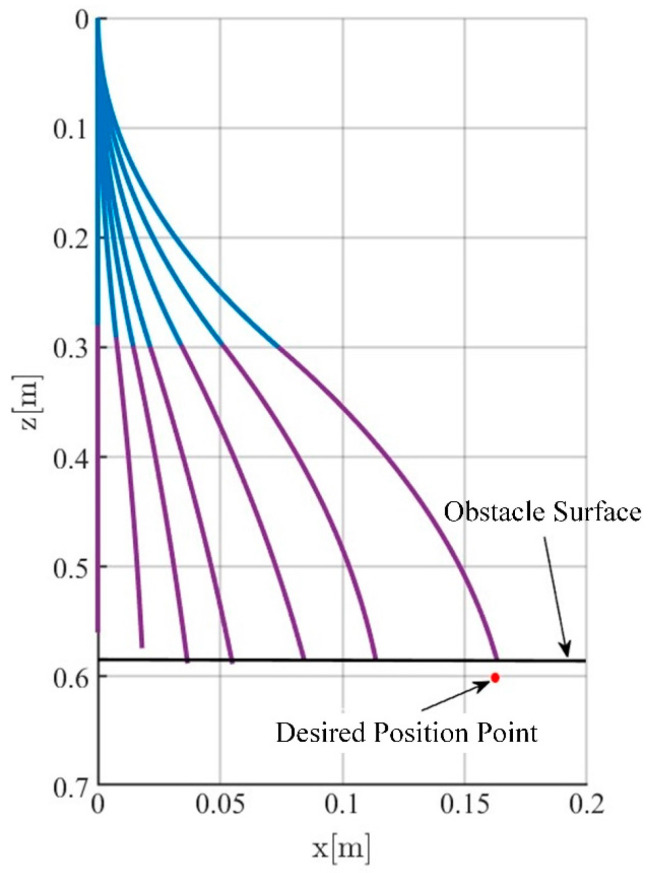
Motion trajectory of soft manipulator when obstacle surface is flat.

**Figure 8:**
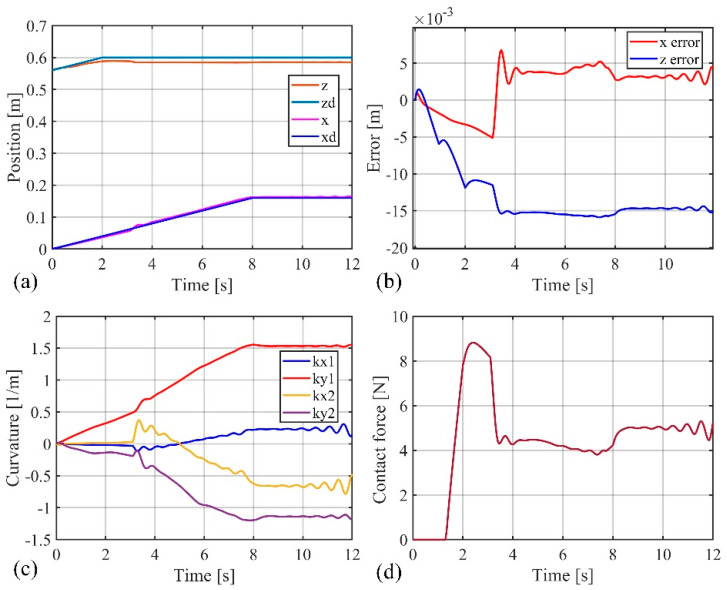
Simulation results when obstacle surface is flat, (**a**) The position change curve of the end of the soft robotic arm in the x and z directions; (**b**) The position error change curve of the end of the soft robotic arm in the x and z directions; (**c**) Curve of curvature variation of soft robotic arm; (**d**) The contact force variation curve of the soft robotic arm end in the x and z directions.

Figure 7 and Figure 8 present the movement trajectory and the variation curves of the soft robotic arm’s end-effector position, when the obstacle surface is set to the z = 0.58 plane and the desired position is set to (0.16 m, 0.6 m). In Figure 8, the stable absolute position error of the soft robotic arm’s end-effector in the x direction is 0.003 m, with a peak absolute error of 0.007 m and relative errors of 2% and 4.3%, respectively. The stable absolute position error in the z direction is −0.015 m, with a peak absolute error of −0.016 m and relative errors of −2.5% and −2.7%, respectively. In addition, the stable contact force in the z direction is 5 N, and the peak contact force is 9 N.

**Figure 9:**
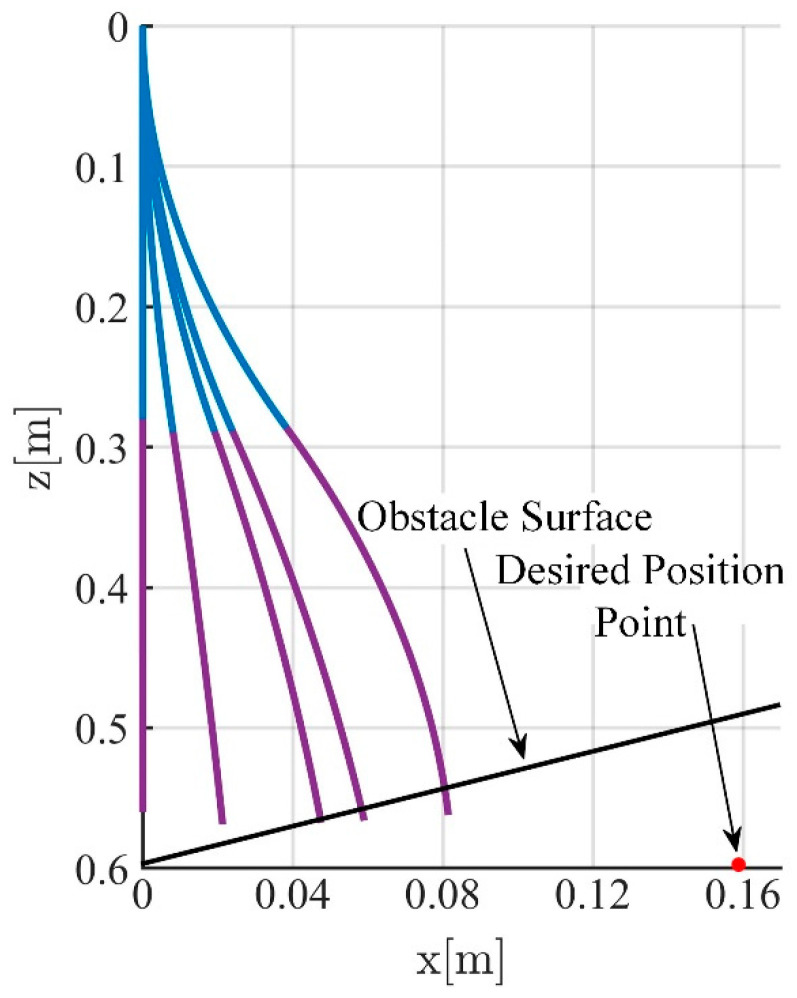
Motion trajectory of soft manipulator when obstacle surface is inclined.

**Figure 10:**
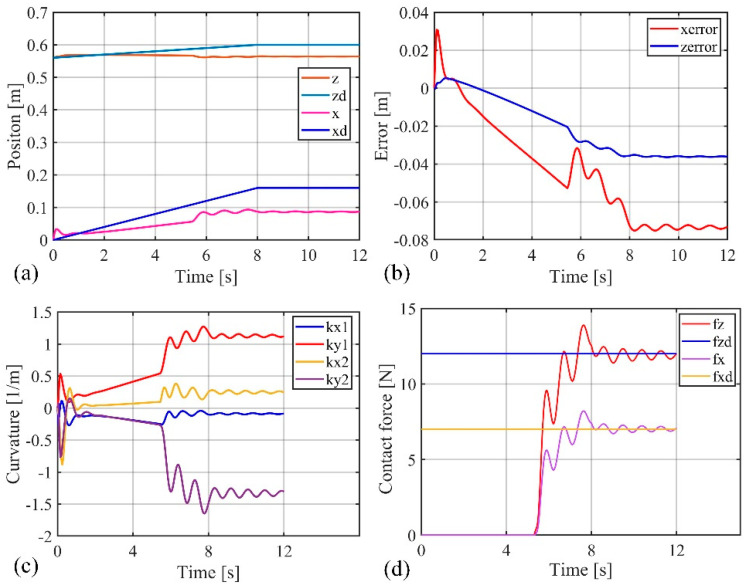
Simulation results when obstacle surface is inclined, (**a**) The position change curve of the end of the soft robotic arm in the x and z directions; (**b**) The position error change curve of the end of the soft robotic arm in the x and z directions; (**c**) Curve of curvature variation of soft robotic arm; (**d**) The contact force variation curve of the soft robotic arm end in the x and z directions.

Figure 9 and Figure 10 present the movement trajectory and the variation curves of the soft robotic arm’s end-effector position, when the obstacle surface is set to an inclined plane (0.6 x + z = 0.6) and the desired position is set to (0.16 m, 0.6 m). In Figure 10, the stable absolute position error in the x direction is −0.075 m, with a peak absolute error of −0.078 m and relative errors of −46.9% and −48.8%, respectively. The stable absolute position error in the z direction is −0.038 m, with a peak absolute error of −0.039 m and relative errors of −6.3% and −6.5%, respectively. In addition, the stable contact force in the x direction is 7 N, and the peak contact force is 8.5 N. The stable contact force in the z direction is 12 N, and the peak contact force is 14 N.

**Figure 11:**
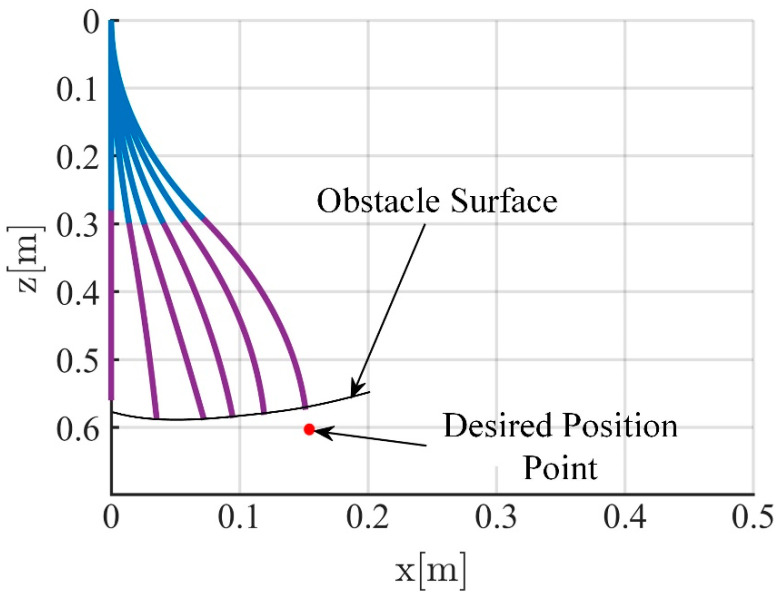
Motion trajectory of soft manipulator when obstacle surface is curved.

**Figure 12:**
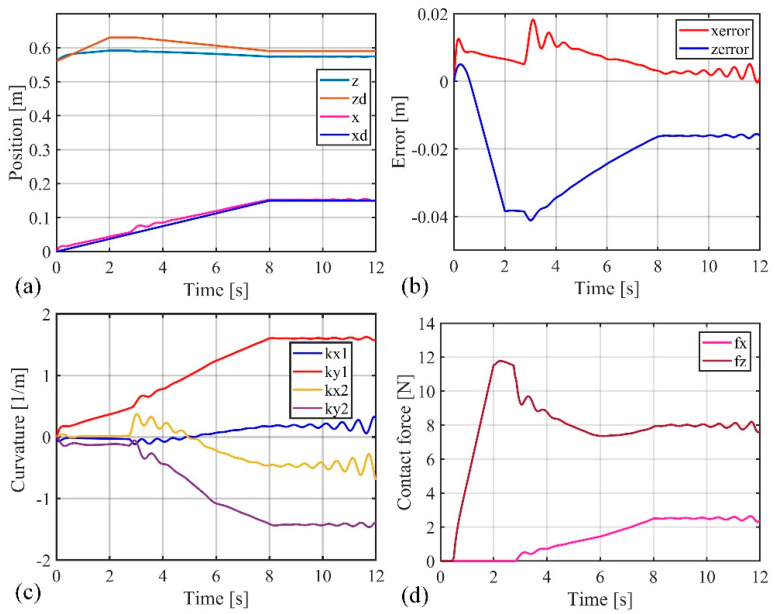
Simulation results when obstacle surface is curved, (**a**) The position change curve of the end of the soft robotic arm in the x and z directions; (**b**) The position error change curve of the end of the soft robotic arm in the x and z directions; (**c**) Curve of curvature variation of soft robotic arm; (**d**) The contact force variation curve of the soft robotic arm end in the x and z directions.

Figure 11 and Figure 12 present the movement trajectory and the variation curves of the soft robotic arm’s end-effector position, when the obstacle surface is set to an arc surface (${\left(x-0.05\right)}^{2}+{\left(z-0.28\right)}^{2}=0.09$) and the desired position is set to (0.16 m, 0.6 m). In Figure 12, the stable absolute position error in the x direction is 0.002 m, with a peak absolute error of 0.018 m and relative errors of 1.25% and 11.3%, respectively. The stable absolute position error in the z direction is −0.016 m, with a peak absolute error of −0.041 m and relative errors of −2.7% and −6.8%, respectively. In addition, the stable contact force in the x direction is 2.5 N, and the peak contact force is 2.8 N. The stable contact force in the z direction is 8 N, and the peak contact force is 11.9 N.

By simulating different obstacle surfaces, the data results show that when the obstacle surface is a plane, the position errors and contact forces are minimal. When the obstacle surface is an arc, they are moderate, and when the obstacle surface is inclined, the position errors and contact forces are the largest. Thus, the active–passive composite impedance controller performs best under the plane obstacle condition, with slightly worse performance under the inclined plane obstacle condition. However, by comprehensively comparing the simulation results under different conditions, it is evident that the active–passive composite impedance controller can control the soft robotic arm to move smoothly under different types of obstacles, with small position errors and contact forces, and stable fluctuations, demonstrating excellent control performance.

## 4. Experimental Analysis

### 4.1. Experimental Platform

**Figure 13:**
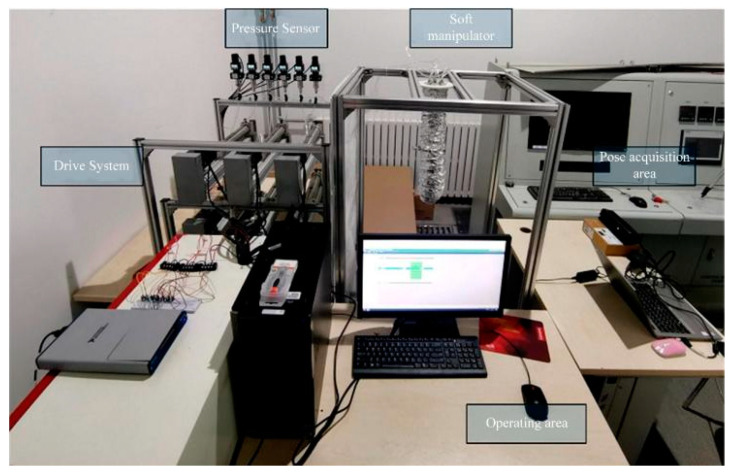
Simulation results when the obstacle surface is curved.

To validate the control performance of the composite active–passive impedance controller designed in this paper in practical applications, an experimental platform was built, as shown in Figure 13. The experimental platform mainly consists of an upper computer, a hydraulic drive system, pressure sensors, and an information acquisition system.

The soft robotic arm used in this paper consists of two soft robotic arm segments connected in series, with each segment comprising three soft units. The movement of the entire arm can be controlled by deforming the six soft units. As shown in Figure 13, the experimental platform includes a hydraulic drive system that can independently drive each soft unit of the soft robotic arm. The hydraulic drive system drives a ball screw by controlling the rotation of a servo motor. The ball screw is tightly connected to the piston rod of the hydraulic cylinder, allowing the servo motor’s rotation to control the telescopic motion of the piston rod. Since the motion relationships between the servo motor, ball screw, and hydraulic cylinder piston rod correspond one-to-one, the piston rod’s extension and retraction can be precisely controlled by adjusting the servo motor’s rotation angle. Each actuator in the hydraulic drive system drives only one soft unit, meaning each hydraulic cylinder is connected to only one soft unit. Therefore, a sealed chamber is formed between the hydraulic cylinder and the soft unit. When the piston rod moves, the fluid in the hydraulic cylinder flows into the soft unit’s cavity through a connecting channel under the piston’s pressure, thus inflating the soft unit. The hydraulic drive system can input the required driving pressure for the soft robotic arm by controlling the displacement of the hydraulic cylinder’s piston rod. Additionally, a marker is attached to the end of the soft robotic arm, and its position is detected by the information acquisition system. The position data are then transmitted to the upper computer to complete the feedback loop for position control.

### 4.2. External Force Impact Experiment

**Figure 14:**
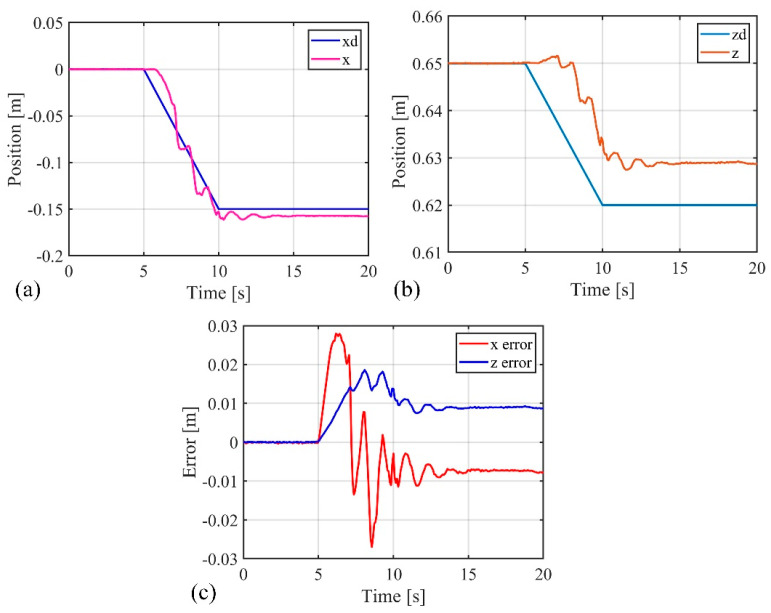
Data curves for position control experiment, (**a**) The position change curve of the end of the soft robotic arm in the x-direction; (**b**) The change curve of the position of the end of the soft robotic arm in the z-direction; (**c**) The error of the end position of the soft robotic arm in the x and z directions.

In the following experiments, the control performance and advantages of the composite active–passive impedance controller designed in this paper were verified. To facilitate the feedback of the soft robotic arm’s end-point position data and contact force, while reducing the demand for feedback sensors, the experiments focused on controlling the soft robotic arm’s movement within the x-o-z plane. First, the active–passive composite impedance controller was used to drive the soft robotic arm toward the desired position, set at (−0.15 m, 0.62 m), without any external contact forces or obstacles influencing the soft robotic arm during the process. The experimental data are shown in Figure 14. From Figure 13, it can be seen that the stable absolute position error of the soft robotic arm’s end point in the x direction is −0.008 m, with a peak absolute error of −0.028 m, and relative errors of 5.3% and 18.7%, respectively. In the z direction, the stable absolute position error is 0.009 m, with a peak absolute error of 0.019 m, and relative errors of 1.5% and 3.1%, respectively. Based on the above data analysis, it can be concluded that when the soft robotic arm is not affected by external contact forces or obstacles during movement, the active–passive composite impedance controller designed in this paper can effectively guide the soft robotic arm to the desired position with minimal position error, demonstrating good control performance.

**Figure 15:**
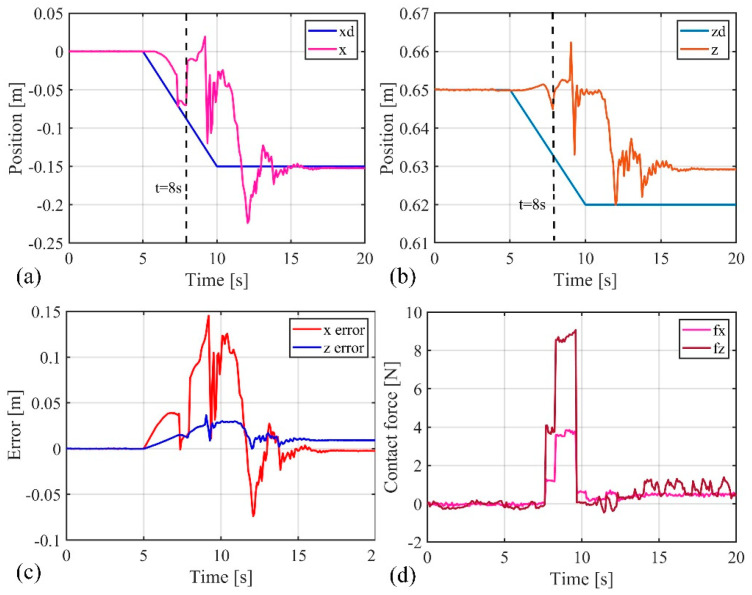
Data curves for external force impact experiment, (**a**) The position change curve of the end of the soft robotic arm in the x-direction; (**b**) The change curve of the position of the end of the soft robotic arm in the z-direction; (**c**) The error of the end position of the soft robotic arm in the x and z directions; (**d**) The contact force variation curve of the soft robotic arm end in the x and z directions.

**Figure 16:**
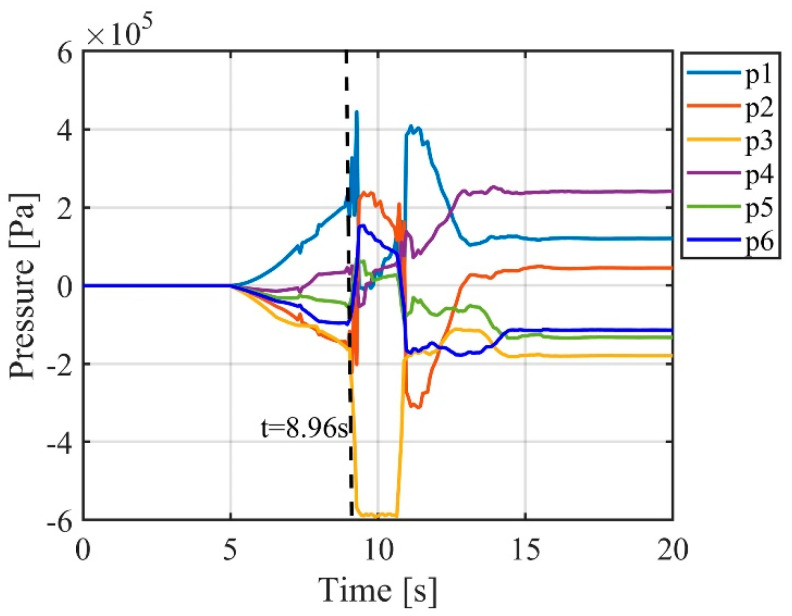
Control input curve for external force impact experiment.

The previous position control experiments demonstrated that the composite active–passive impedance controller can achieve precise position control of the soft robotic arm. In the following, an impact force experiment was conducted to verify the controller’s ability to adapt to external disturbances. The soft robotic arm was controlled to move toward the desired position using the active–passive composite impedance controller, with the target position set at (−0.15 m, 0.62 m). During the motion, an external force was suddenly applied to the soft robotic arm’s end point to disturb its movement, which was then removed. The experimental results are shown in Figure 15 and Figure 16. From Figure 15, it can be observed that the stable absolute position error of the soft robotic arm’s end point in the x direction is 0 m, with a peak absolute error of 0.14 m, and relative errors of 0% and −93.3%. In the z direction, the stable absolute position error is 0.01 m, with a peak absolute error of 0.04 m, and relative errors of 1.6% and 6.5%. Additionally, the peak contact force in the x direction is 4 N, and in the z direction, it is 9 N. Once the external force disappears, the contact force quickly tends toward 0. From the above data analysis, it is evident that when the active–passive composite impedance controller controls the soft robotic arm and is suddenly subjected to external force, the soft robotic arm exhibits impedance characteristics under the controller’s influence. This results in position errors and contact forces, with a trade-off in position control accuracy to prevent large contact forces, allowing the soft robotic arm to exhibit compliance to external impacts, thus avoiding damage. Once the external force is removed, the contact force rapidly returns to 0, and the soft robotic arm quickly moves toward the initial desired position under the controller’s influence, ultimately reaching the desired position with the position error approaching 0. From the above analysis, it can be concluded that the active–passive composite impedance controller continues to exhibit good control performance in harsh environments, such as under external impacts, indicating its robust control capability.

Additionally, as mentioned earlier, the advantages of soft robotic arms over rigid robots in impedance control were highlighted. In this experiment, the advantages of the passive impedance characteristics of the soft robotic arm were verified. By comparing Figure 15 and Figure 16, it can be seen that when the soft robotic arm is subjected to an external force, the monitored position and contact force data of the soft robotic arm’s end point exhibit a sudden change at 8 s, while the control input of the soft robotic arm (i.e., the output pressure of the controller) changes at 8.96 s. These results indicate that when the soft robotic arm suddenly makes contact with the external environment, the monitored position of the soft robotic arm’s end point changes instantaneously at the moment of contact. However, the control input adjusted by the impedance controller only changes after a short delay, to adjust the impedance effect. Therefore, the passive impedance characteristics of the soft robotic arm respond faster than the impedance controller, and when the soft robotic arm collides with the external environment, it does not damage the arm, providing higher safety. In impedance control of rigid robots, the response speed to the environment depends on the impedance controller’s response speed. When a rigid robot is subjected to an external impact, if the impedance controller responds slowly and cannot adjust the control input in real time to track environmental changes, the rigid robot will collide rigidly with the environment, which may severely damage the robot, leading to lower safety. Additionally, the use of the impedance controller requires contact force feedback, which places higher demands on sensors. For example, in this study, a force sensor was fixed at the soft robotic arm’s end, so contact force was only generated when the end of the soft robotic arm interacted with the environment, and the impedance controller is only effective in this case. The impedance controller has more limitations on its effectiveness. In contrast, the passive impedance characteristics of the soft robotic arm exist throughout its body, and regardless of which part of the soft robotic arm comes into contact with the environment, its passive impedance properties will act to prevent rigid collisions and avoid damaging the soft robotic arm. Due to the significant advantages of the soft robotic arm’s passive impedance characteristics, the passive impedance characteristics are introduced into the design of the active–passive composite impedance controller for the soft robotic arm, which also offers advantages such as fast response, good controllability, and high safety.

### 4.3. Obstacle Surface Following Experiment

In the previous section, it was experimentally demonstrated that the active–passive composite impedance controller maintains good performance even in harsh environments with external impacts, and the advantages of the soft robotic arm’s inherent passive impedance characteristics were also highlighted. In this section, an obstacle surface-following experiment was conducted to verify the control performance of the active–passive composite impedance controller when in contact with an obstacle surface.

**Figure 17:**
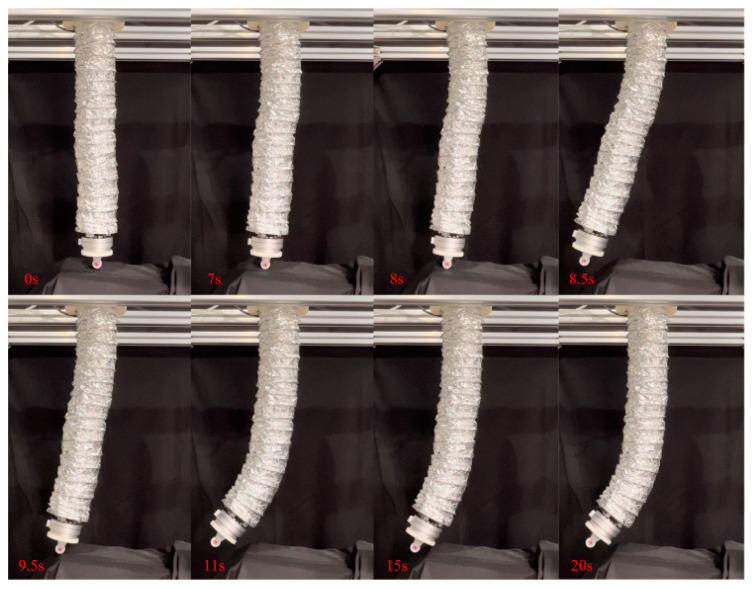
Actual motion process of soft robotic arm on a short, inclined surface.

**Figure 18:**
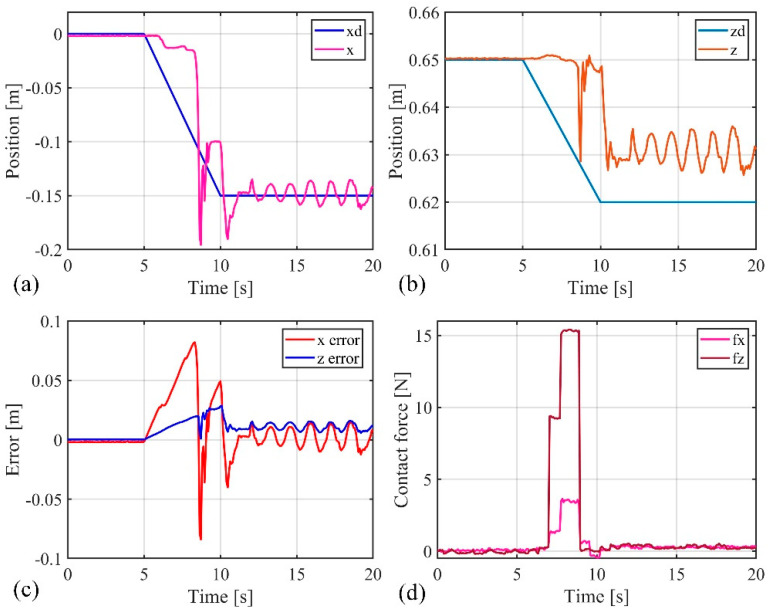
Experimental data curve when in contact with short obstacle, (**a**) The position change curve of the end of the soft robotic arm in the x-direction; (**b**) The change curve of the position of the end of the soft robotic arm in the z-direction; (**c**) The error of the end position of the soft robotic arm in the x and z directions; (**d**) The contact force variation curve of the soft robotic arm end in the x and z directions.

First, the active–passive composite impedance controller was used to drive the soft robotic arm towards the desired position, which was set at (−0.15 m, 0.62 m). A short, inclined obstacle surface was placed to ensure that the soft robotic arm would come into contact with the obstacle surface during its movement towards the desired position, then detach after a brief contact. The motion process of the soft robotic arm during the short obstacle surface following experiment is shown in Figure 17. The experimental results are shown in Figure 18. From the figure, it can be observed that the soft robotic arm’s end effector has a stable absolute error of 0 m in the x direction, with a peak absolute error of −0.08 m, and relative errors of 0% and 53.3%. In the z direction, the stable absolute error is 0.01 m, with a peak absolute error of 0.025 m, and relative errors of 1.6% and 4%. Additionally, the peak contact force in the x direction is 3.5 N, and in the z direction, the peak contact force is 15.5 N. Once the soft robotic arm detaches from the obstacle surface, the contact force rapidly returns to zero. From the above data analysis, it can be concluded that after the soft robotic arm makes contact with the inclined obstacle surface, the active–passive composite impedance controller ensures that the soft robotic arm continues to move along the obstacle surface without getting stuck. The dynamic relationship between the soft robotic arm’s end position and the contact force ensures smooth motion without generating excessive contact force. After the soft robotic arm detaches from the obstacle surface, the controller can still guide the soft robotic arm along the initial desired trajectory, ultimately reaching the target position.

**Figure 19:**
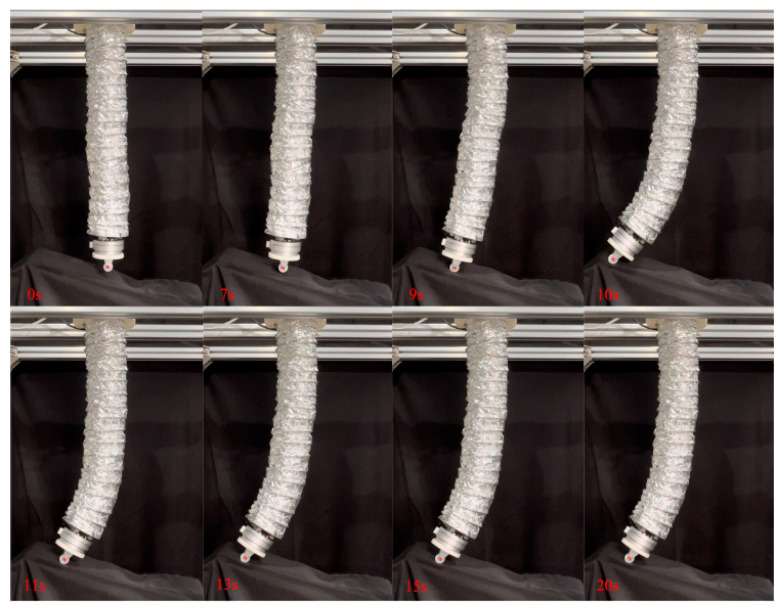
Actual motion process of soft robotic arm on a long, inclined surface.

**Figure 20:**
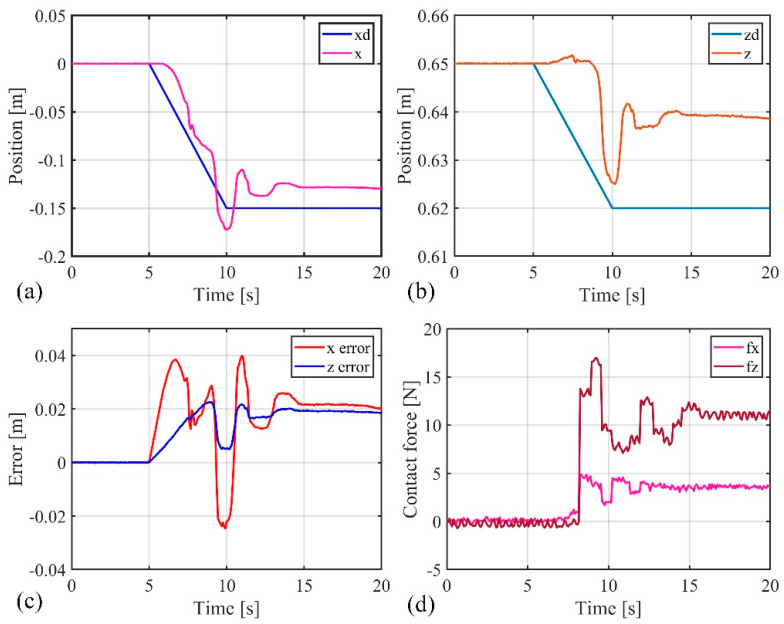
Experimental data curve when in contact with long obstacle, (**a**) The position change curve of the end of the soft robotic arm in the x-direction; (**b**) The change curve of the position of the end of the soft robotic arm in the z-direction; (**c**) The error of the end position of the soft robotic arm in the x and z directions; (**d**) The contact force variation curve of the soft robotic arm end in the x and z directions.

Next, the active–passive composite impedance controller was used to guide the soft robotic arm towards the target position, which was set again at (−0.15 m, 0.62 m), and a longer inclined obstacle surface was arranged so that the arm maintained continuous contact with it during movement. The motion process of the soft robotic arm during the long obstacle surface following experiment is shown in Figure 19. The experimental results are shown in Figure 20. The figure shows that in the x direction, the end of the soft robotic arm has a stable absolute error of 0.025 m and a peak absolute error of 0.04 m, with relative errors of −16.7% and −26.7%. In the z direction, the stable absolute error is 0.02 m, with a peak absolute error of 0.025 m and relative errors of 3.2% and 4%. Additionally, the stable contact force in the x direction is 3 N, with a peak contact force of 5 N; in the z direction, the stable contact force is 11 N, and the peak contact force is 17 N. Analysis of these data indicates that, once in contact with the obstacle surface, the soft robotic arm exhibits the desired impedance characteristics under the control of the composite impedance controller. This ensures that the arm can move continuously along the obstacle surface without becoming stuck. The dynamic relationship established between the soft robotic arm’s endpoint position and contact force allows smooth movement without generating excessive contact force.

**Figure 21:**
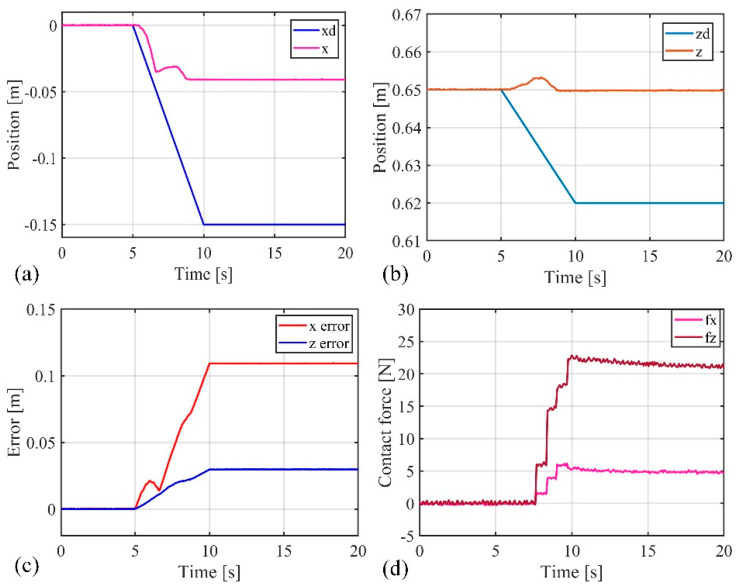
Experimental data curve of pure passive impedance, (**a**) The position change curve of the end of the soft robotic arm in the x-direction; (**b**) The change curve of the position of the end of the soft robotic arm in the z-direction; (**c**) The error of the end position of the soft robotic arm in the x and z directions; (**d**) The contact force variation curve of the soft robotic arm end in the x and z directions.

To further verify the advantages of the proposed active–passive composite impedance controller over the purely passive impedance of the soft robotic arm, an experiment using a sliding mode position controller developed in prior work was conducted. The target position was again set to (−0.15 m, 0.62 m), with a similarly inclined long obstacle surface, as used in the previous impedance test. The results shown in Figure 21 indicate that in the x direction, the soft robotic arm’s endpoint has a stable absolute error of 0.11 m, with a relative error of 73.3%. In the z direction, the stable absolute error is 0.03 m, with a relative error of 4.8%. Additionally, the stable contact force is 5 N in the x direction and 21 N in the z direction. Analysis of these data reveals that, when the sliding mode position controller guides the soft robotic arm in contact with the inclined obstacle, the arm’s passive impedance characteristics prevent rigid collision with the obstacle, thus avoiding damage. However, due to the lack of control over the arm’s passive impedance, contact with the obstacle causes positional errors to increase, which in turn amplifies the controller’s input. As the controller input becomes insufficient to overcome the obstacle, the contact force continues to grow, ultimately causing the arm to become stuck and preventing it from reaching the target position. Comparing Figure 20 and Figure 21 demonstrates that under identical conditions, the active–passive composite impedance controller achieves smaller position errors and contact forces and prevents the arm from becoming stuck due to obstacle interference. This controller offers improved control over impedance characteristics and greater adaptability to environmental challenges.

The experimental results demonstrate that the proposed active–passive composite impedance controller combines the advantages of active–passive composite impedance with the soft robotic arm’s intrinsic passive impedance. This approach not only maintains control over impedance characteristics but also enhances response speed, ensuring that the soft robotic arm moves smoothly and compliantly when contacting obstacles. It avoids getting stuck and minimizes contact forces, reducing the risk of damage to the soft robotic arm.

## 5. Conclusions

This article introduces an active–passive composite impedance controller and verifies its control performance in soft robotic arm control. Firstly, an active–passive composite impedance controller based on force type impedance control was developed by considering the passive impedance characteristics of the soft robotic arm. In addition, a location-based impedance controller was designed to compare the performance of active–passive composite impedance controllers, and simulations were conducted for comparison. The simulation results indicate that the active–passive composite controller effectively combines the controllability of active impedance with the advantages of passive impedance of the soft robotic arm. Finally, to verify the actual control performance of the soft robotic arm active–passive composite impedance controller, an experimental platform was built for impact and obstacle surface following tests. The experimental results of the active–passive impedance controller show that when encountering obstacles or external forces, the soft robotic arm will move smoothly and obediently, minimizing excessive contact force and reducing the risk of system damage. Furthermore, the controller also exhibits reduced position error, faster response time, and strong adaptability to environmental changes. The experimental results confirm that the controller maintains strong control performance and adaptability in complex scenarios involving external impacts and different obstacles. This design provides new possibilities for the application of soft robots in complex environments under contact constraints. The learning capacity of neural networks can be exploited to optimize the adaptability of the active–passive composite impedance controller for soft robotic arms to complex environments in the future.

## Figures and Tables

**Table 1:** Parameters of the soft manipulator and the hydraulic drive system.

Symbol	Name	Parameter Values
*l_0_*	Initial length of soft unit	280 mm
*d_a_*	Distance from the center of the soft unit to the center of the soft arm	30 mm
*d_c_*	Soft unit cavity radius	15 mm
*d_e_*	Soft arm radius	50 mm
*d_f_*	Soft unit wall thickness	5 mm
*E*	Elastic modulus	1.2 MPa
*G*	Shear modulus	405 kPa
*I*	Sectional moment of inertia of soft arm	8.0 × 10^−7^ m^4^
*m*	Single section soft arm quality	1.25 kg
*A_d_*	Internal cavity cross-sectional area of soft unit	3.14 × 10^−4^ m^2^
*A_s_*	Circular solid cross-sectional area of soft unit	3.93 × 10^−4^ m^2^
*A_c_*	Effective working area of the piston rod on one side of the rod	2.1 × 10^−3^ m^2^
*V*_0_	Initial volume of liquid before compression in hydraulic drive system	8.135 × 10^−4^ m^3^
*K*	Liquid bulk modulus of elasticity	2400 MPa

## Data Availability

Data are contained within the article.
